# Growing Trends in Scientific Publication in Physiotherapy Treatment of Knee Osteoarthritis: A Bibliometric Literature Analysis

**DOI:** 10.7759/cureus.48292

**Published:** 2023-11-05

**Authors:** Medhavi V Joshi, Chaitanya A Kulkarni, Om C Wadhokar, Mayur B Wanjari

**Affiliations:** 1 Musculoskeletal Sciences, Dr. D. Y. Patil College of Physiotherapy, Pune, IND; 2 Community Based Rehabilitation, Dr. D. Y. Patil College of Physiotherapy, Pune, IND; 3 Public Health, Jawaharlal Nehru Medical College, Datta Meghe Institute of Higher Education and Research, Wardha, IND; 4 Research and Development, Jawaharlal Nehru Medical College, Datta Meghe Institute of Higher Education and Research, Wardha, IND

**Keywords:** exercise therapy, ultrasound, star excursion balance test, otago exercises, phonophoresis, knee osteoarthritis

## Abstract

There has been an increase in the life expectancy of people worldwide, especially in developing countries like India. Osteoarthritis, a condition that usually onsets during later decades of life, has also been on the rise, even with advancing technology. This has led osteoarthritis of the knee to become a global disabling condition of the lower extremity that increases dependency on the affected individual. A bibliometric study has not been conducted on knee osteoarthritis research. Therefore, a bibliometric analysis which includes statistical analysis of recent articles, books, and other forms of publications is done for evaluation of scientific output and to find the importance of scientific studies in terms of quality as well as quantity. The aim of this analysis was to evaluate the productivity of research articles indexed in PubMed related to the condition. The PubMed database was used and articles related to osteoarthritis of the knee, phonophoresis, and start excursion balance test were extracted. In the bib text format, all the files were downloaded and placed together. The R studio software (R Foundation for Statistical Computing, Vienna, Austria) for bibliometric analysis was then used, into which the research data was uploaded and a data framework of bibliometric analysis was made. Analysis of bibliometric publications related to knee osteoarthritis, phonophoresis, Otago exercises, star excursion balance test, ultrasound, and exercise therapy generated between 1989 and 2021 lists a total of 120 relevant documents from 75 sources with an average of 4.53 articles per year of publication. The use of an advanced PubMed database enables the extraction of adequate articles and powerful bibliometric analysis of the studies conducted on osteoarthritis of the knee published from 1989 to 2021. It includes an assessment of the contributions from major countries. This study allowed us to validate our methodology which can be used to evaluate research policies and promote international collaboration.

## Introduction and background

Bibliometric analysis helps to identify and trace various intellectual structures in the specific research field. It helps in identifying and then generating patterns, for developing more structured literature reviews that include relevant information. For the purpose of this study, the topic under research is osteoarthritis of the knee. Osteoarthritis of the knee is a progressive degenerative condition prevailing either unilaterally or bilaterally. It leads to a decline in the independence of the affected individual, making them disabled [1]. Osteoarthritis has many factors leading to its onset at an early age. Obesity and diabetes have been found to be major causes of early-onset knee arthritis because of its rising prevalence in developed countries. Another factor that is responsible for the rising incidence of osteoarthritis is the overall increase in life expectancy in developing countries due to advancements in medical technology, for prevention as well as curation [2]. Considering the escalation in the number of cases of osteoarthritis today, even with advanced medical technology, this research study aims to gather information about every aspect of the topic of interest [3,4]. Along with surgeries, an alternative approach for the treatment of osteoarthritis was the focus of this research for the benefit of public health globally [5,6]. Bibliometric analysis is one such tool where qualitative and quantitative statistical analysis of all the articles and of all types, is carried out [7,8]. Since very few bibliometric studies are available, we, through this study, aim to design a bibliometric framework of all the relevant literature [9-11].

## Review

Methodology

The aim of this study was to evaluate both the qualitative and quantitative productivity of articles indexed in PubMed, Web of Science and Scopus using Bibliometric R studio software (R Foundation for Statistical Computing, Vienna, Austria), a free-to-use software. A total of six keywords, which are "osteoarthritis of knee", "phonophoresis", "Otago exercises", "star excursion balance test", "ultrasound", and "exercise therapy", were used to retrieve all the relevant articles to date. Keywords were chosen to target only the specific aspects related to osteoarthritis like balance and pain management using electrotherapy modalities and exercise protocol. In the domain of the type of document, we have selected original articles, systemic reviews, meta-analyses, books, clinical trials, and randomized control trials. The language of writing was selected to be English. The bibliometric framework analysis contains information about the authors and their affiliations, the type of documents, citations, and keywords. The software generates data with graphical representations and a bib.txt file is created. The data is then interpreted to form a research article to systematically represent the findings.

Result

Analysis of Document and Authors

**Figure 1:**
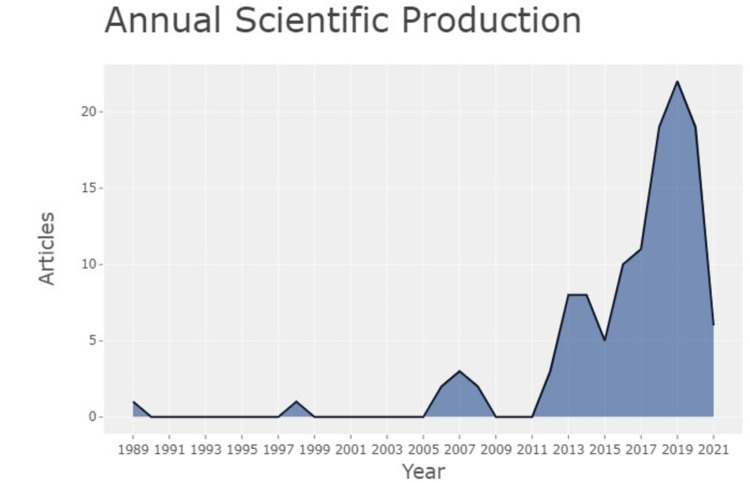
Annual scientific production X-Axis: Years Y-Axis: Number of articles Credit: Image created by the author

A list of 120 documents from 75 sources of research articles associated with osteoarthritis of the knee, phonophoresis, exercise therapy, ultrasound, Otago exercise, and star excursion balance test available in the PubMed database from 1989 to 2021, was generated. A total of 683 author list was generated. The proportion of authors having published a single article is 0.890 (total number = 608) and those with two published articles are 0.095. The total number of authors with three publications was found to be nine and their proportion is 0.013. The highest number of articles were published in the year 2019 with the number being 25; whereas, the lowest number of relevant articles published was close to one or none between 1991 and 1995. Progress in the yearly growth of research articles is clearly seen (Figure 1).

**Figure 2:**
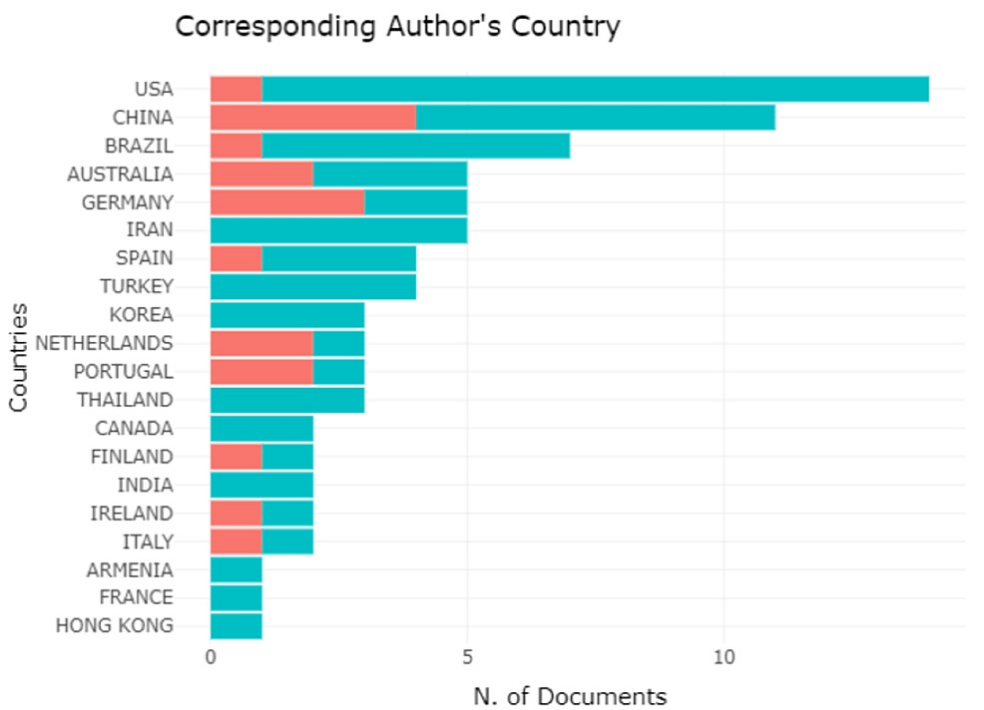
Corresponding author’s country X-Axis: Number of documents Y-Axis: Countries of the corresponding author Green denotes a single corresponding author and red denotes multiple corresponding authors Credit: Image created by the author

There are more publications from the United States of America, China, and Brazil followed by Australia, Germany, Iran, and Spain and fewer than five articles are from Turkey, Korea, Netherlands, etc. (Figure 2).

Analysis of the Journal and Its Growth Trend

**Figure 3:**
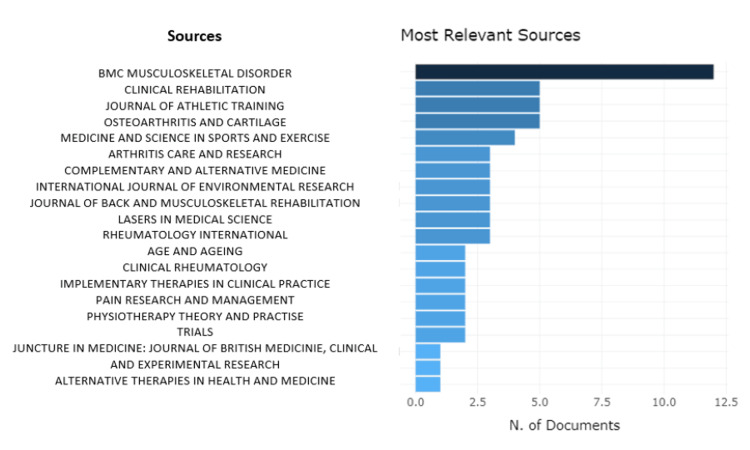
Most relevant sources with the number of documents X-Axis: Number of documents Y-Axis: Sources Credit: Image created by the author BMC: British Medical Council

**Figure 4:**
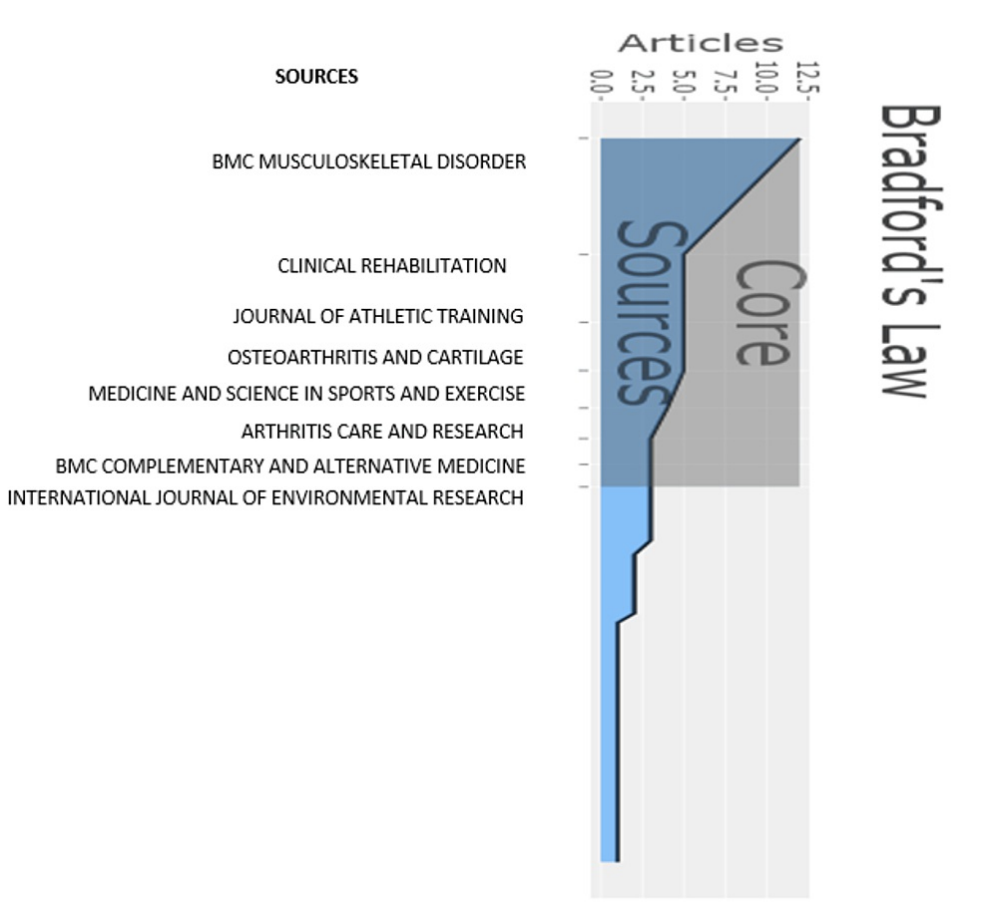
Bradford’s law representing the core sources of research Credit: Image created by the author BMC: British Medical Council

The bibliometric scientific analysis generated a hierarchical list of sources of published research articles. The British Medical Council (BMC) Musculoskeletal Journal was the most relevant with 12 articles along with Clinical Rehabilitation, Journal of Athletic Training and Osteoarthritis, and Cartilage Journal with five articles each. Journal with only one relevant research article for our study was Alternative Therapies in Health and Medicine, Aging Clinical and Experimental Research, and Journal of the British Medical Acupuncture Society (Table 1, Figures 3, 4).

**Table 1:** Tabular representation of sources of research articles BMC: British Medical Council

Sources	Articles
BMC Musculoskeletal Disorders	12
Clinical Rehabilitation	5
Journal of Athletic Training	5
Osteoarthritis and Cartilage	5
Medicine and Science in Sports and Exercises	4
Arthritis Care and Research	3
BMC Complementary and Alternative Medicine	3
International Journal of Environmental Research and Public Health	3
Journal of Back and Musculoskeletal Rehabilitation	3
Laser in Medical Sciences	3
Rheumatology International	3
Age and Ageing	2
Clinical Rheumatology	2
Complimentary Therapies in Clinical Practice	2
Pain Research and Management	2
Physiotherapy Theory and Practice	2
Trials	2
Acupuncture in Medicine	1
Ageing Clinical and Experimental Research	1
Alternative Therapies in Health and Medicine	1

Analysis of Research Database According to Bradford’s Law

Bradford’s law analysis gives us the number of core journals and Bradford’s multiplier that a researcher requires when an additional dozen articles are needed for the purpose of the study. The graphical representation of the core journal when analyzed by Bradford’s law (Figure 4).

Analysis of Institutions and Countries

**Figure 5:**
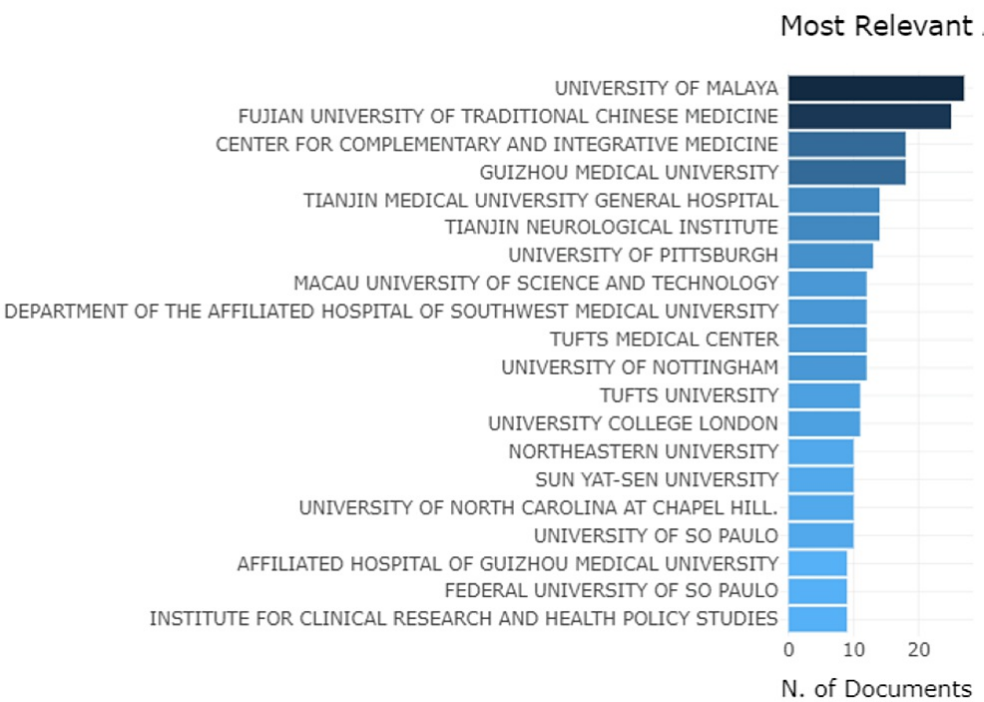
Graphical representation of universities which had the most affiliations Credit: Image created by the author

The most relevant affiliations were from the University of Malaya and Fujian University of Traditional Chinese Medicine followed by the Center for Complimentary and Integrative Medicine and Guizhou Medical University (Figure 5).

**Figure 6:**
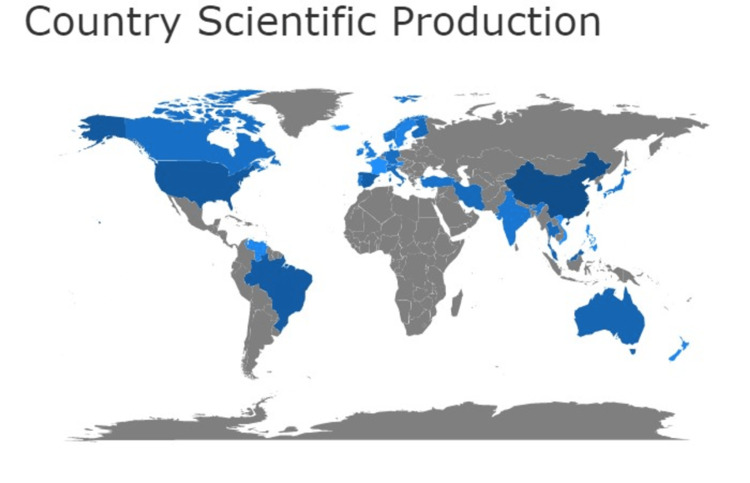
Map representing the scientific production of the country Credit: Image created by the author

The pictorial representation of the scientific production of the country shows a high number of researchers from China, the United States, and Brazil denoted with dark blue followed by countries like Australia, India, and Canada (Figure 6).

Analysis of Citations and Author's Publications

**Figure 7:**
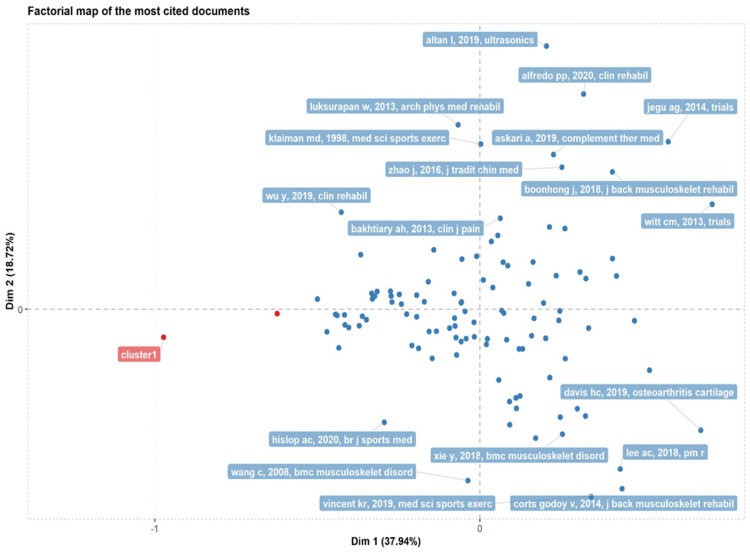
Factorial representation of the most cited documents Credit: Image created by the author

The knowledge of cited articles and their author’s publications over the years is an important aspect when collecting scientific literature. The articles showing the highest number of citations reflect its quality. The use of such articles for evidence-based practice makes it of great clinical importance (Figure 7).

**Figure 8:**
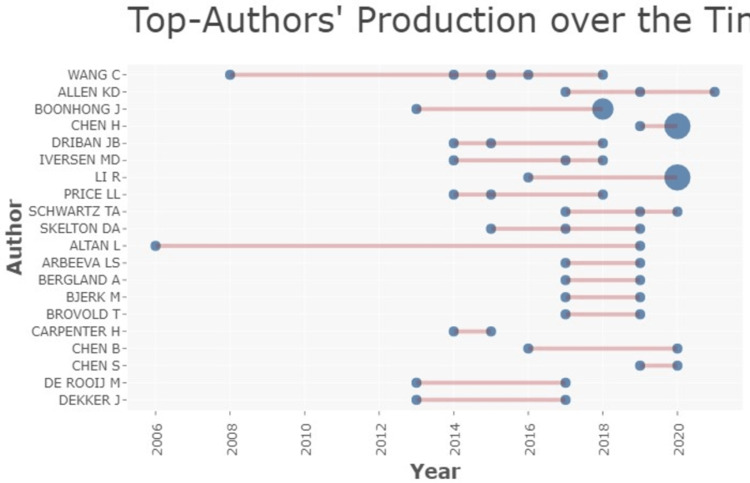
Top author's production over time Credit: Image created by the author

The top author’s production of research articles over a period of fourteen years, from 2006 and 2020 is represented by the graph below. Wang is at the top of the hierarchical list (Figure 8).

Analysis of Words

**Figure 9:**
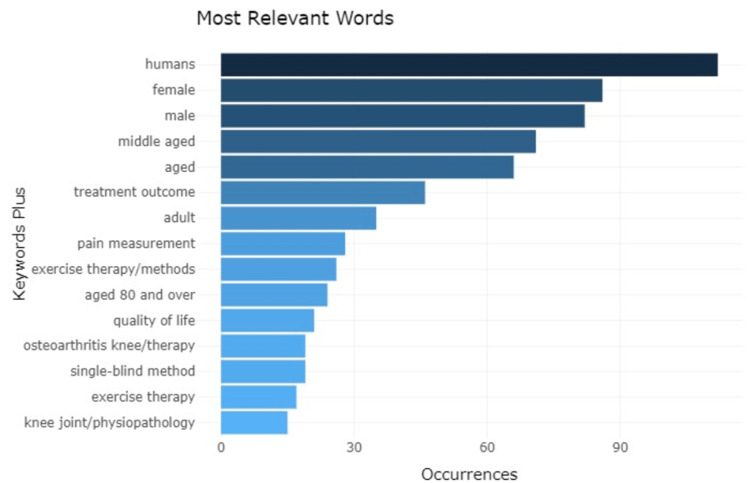
Depiction of most relevant words and their frequency from the research articles Credit: Image created by the author

**Figure 10:**
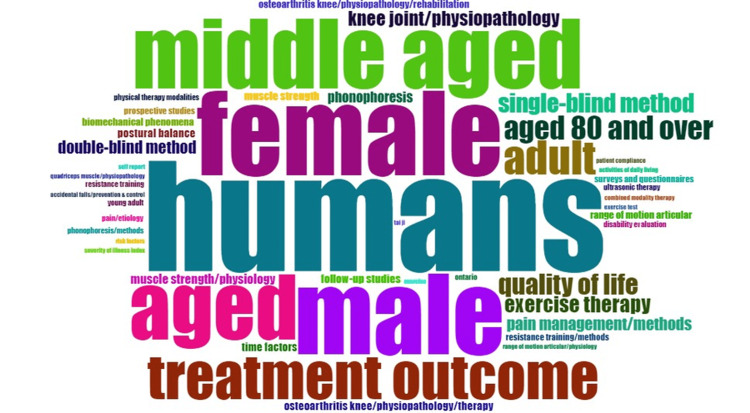
Most common keywords from the study Credit: Image created by the author

The study reveals that the most frequently used word is human with a frequency of 112 followed by male, female, and middle-aged with a frequency of 86, 82, and 71, respectively. The word exercise therapy was used the least with a frequency of 26 (Figures 9, 10).

A wide range of networks exists between the most frequently used words globally among all the research articles and is depicted by the pictorial representation obtained from the study.

Analysis of the Study Concept and Trends

The conceptual structural map shows the domains and different aspects of the studies conducted on osteoarthritis of the knee, Otago exercise programed, exercise therapy, and the other relevant keywords used for the bibliometric statistical analysis.

Discussion

This analysis through the bibliometric tool of the worldwide research in the field of knee osteoarthritis and its related physiotherapy treatment denoted an increase in the number of articles published in the period of last 25 years [12,13]. This rise in the research articles when compared to other medical fields even though is less, is a trend that shows a rise in the overall research being conducted [14-16].

A total of 683 author lists were generated, with 608 authors publishing a single document, for a proportion of 0.89 [17-19]. The most productive author had a maximum of five articles. The 0.095 was the proportion of authors from the list generated, having two articles only [20,21].

The most relevant articles were from BMC Musculoskeletal Disorder, with 12 articles. The journals with three relevant articles were Arthritis Care and Research, BMC Complementary and Alternative Medicine, International Journal of Environmental Research and Public Health, Laser in Medical Sciences, and Rheumatology International [22,23]. The journals Age and Aging, Clinical Rheumatology, Complimentary Therapies in Clinical Practice, Pain Research and Management, and Physiotherapy Theory and Practice each had two articles of importance. The majority of articles found were on the relationship between different exercise therapies and quality of life in individuals subjected to osteoarthritis of the knee [24,25]. Other articles included the use of ultrasound, an electrotherapeutic modality as an intervention for the treatment of knee osteoarthritis. A comparison of using therapeutic ultrasound and phonophoresis was also part of a few studies [26-28].

A randomized control trial examined the effect of Phyllanthus phonophoresis on knee osteoarthritis and concluded that it was significant in reducing symptomatic pain and improving the results of the six-minute walk test [29]. A similar study conducted in 2015 showed phonophoresis had a significant effect on both, Chinese herbal medicine as well with sodium diclofenac [30]. A study in 2022 showed that Zingiber, an alternative medicine with anti-inflammatory properties was useful in reduction in pain [31].

## Conclusions

This bibliometric study shows the growing trend in the field of treatment for the long-standing condition of the knee, that is osteoarthritis. It showed the shift from leaning toward surgical treatment as a first line of management to exercise therapy being the first line of treatment. The research also concludes that a number of alternative forms of medicine are been used and are under research study to check their efficacy on symptoms of knee osteoarthritis. Therefore, in the field of rehabilitation even though knee osteoarthritis has been under study for decades, research for alternate and more effective forms of treatment for making the condition less dependent and disabling is required.
